# Effects of Food Contamination on Gastrointestinal Morbidity: Comparison of Different Machine-Learning Methods

**DOI:** 10.3390/ijerph16050838

**Published:** 2019-03-07

**Authors:** Qin Song, Yu-Jun Zheng, Jun Yang

**Affiliations:** 1 Scientific Research Institute, Hangzhou Normal University, Hangzhou 311121, China; 2 Institute of Service Engineering, Hangzhou Normal University, Hangzhou 311121, China; yujun.zheng@computer.org; 3 School of Public Health, Zhejiang University, Hangzhou 310009, China; gastate@zju.edu.cn

**Keywords:** food contamination, public health, gastrointestinal diseases, morbidity, deep neural networks, evolutionary learning

## Abstract

Morbidity prediction can be useful in improving the effectiveness and efficiency of medical services, but accurate morbidity prediction is often difficult because of the complex relationships between diseases and their influencing factors. This study investigates the effects of food contamination on gastrointestinal-disease morbidities using eight different machine-learning models, including multiple linear regression, a shallow neural network, and three deep neural networks and their improved versions trained by an evolutionary algorithm. Experiments on the datasets from ten cities/counties in central China demonstrate that deep neural networks achieve significantly higher accuracy than classical linear-regression and shallow neural-network models, and the deep denoising autoencoder model with evolutionary learning exhibits the best prediction performance. The results also indicate that the prediction accuracies on acute gastrointestinal diseases are generally higher than those on other diseases, but the models are difficult to predict the morbidities of gastrointestinal tumors. This study demonstrates that evolutionary deep-learning models can be utilized to accurately predict the morbidities of most gastrointestinal diseases from food contamination, and this approach can be extended for the morbidity prediction of many other diseases.

## 1. Introduction

In recent decades, industrial emissions, domestic waste, and the overuse of pesticides and fertilizers have caused serious environmental pollution, which has been confirmed as an important factor causing alarming deterioration in public health [1,2,3,4,5]. In particular, food contamination arising from soil and water pollution has been reported to be involved in almost all types of gastrointestinal diseases [6,7,8]. However, modeling the effects of food contamination on gastrointestinal morbidity is still a challenging task because the pathogenic mechanisms of gastrointestinal diseases are very complex, the number of contaminants is large, and the pathogenic roles of contaminants in the diseases are often unknown or uncertain.

There are numerous studies on the effects of environmental pollution on public health. A majority of studies have been devoted to the relationships between air pollution and respiratory diseases. Using logistic regression and weighted linear regression, Zhang et al. [9] examined the association between children’s respiratory morbidity prevalence and district-specific ambient levels of main air pollutants in four Chinese cities, and their results evidenced that morbidity prevalence was positively associated with the levels of NO_x_, SO${}_{2}$, and coarse particles. Jayaraman and Nidhi [10] used a generalized additive Poisson regression model to evaluate the association between air pollutants and daily variations in respiratory morbidity in Delhi in 2004–2005. Based on a log-linear Poisson regression model, Sousa et al. [11] performed time-series analysis to assess the impact of air pollution on emergency hospitalization for respiratory disease in Rio de Janeiro, Brazil, in 2000–2005. Zhao et al. [12] used a time-series model with a quasi-Poisson link to examine the association between PM pollution and respiratory morbidities in Dongguan City, China, in 2013–2015. Qiu et al. [13] used a similar approach to estimate the short-term effects of ambient air pollutants (PM${}_{10}$, PM${}_{2.5}$, NO${}_{2}$, and SO${}_{2}$) on hospital admissions of overall and cause-specific respiratory diseases in 17 cities of Sichuan Province, China, during 2015–2016. Although such regression models can demonstrate the associations between pollution and diseases, they are often incapable of providing sufficiently accurate morbidity prediction for healthcare management.

To overcome the limitation of classical linear and logistic models with multiple variables to handle the multifactorial effect, Bibi et al. [14] used an artificial neural network (ANN) to predict the effect of atmospheric changes on emergency department visits for respiratory symptoms. The results showed that the average prediction error of the ANN was much less than the classical models on the test set. Wang et al. [15] applied the Granger causality method to identify the main air pollutants correlated with the mortality of respiratory diseases, and then constructed an ANN model for respiratory mortality prediction in Beijing during 2005–2008, which also achieved higher accuracy than classical correlation-analysis methods. Junk et al. [16] used an ANN to predict the mortality rates of respiratory diseases associated with air pollution under different weather conditions in Western Europe. Moustris et al. [17] developed an ANN model to predict the weekly number of childhood asthma admission at the greater Athens area in Greece from ambient air-pollution data during 2001–2004. Zhu et al. [18] studied the effects of air pollutants on lower respiratory disease in Lanzhou City, China, during 2001–2005, and constructed an ANN based on a group method of data handling to forecast the number of patients in a hospital. Sundaram et al. [19] developed an Elman neural network to predict respiratory mortality and cardiovascular mortality from a set of air-pollution indicators, and the results showed that the dynamic ANN showed good performance on time-series prediction. Recently, Liu et al. [20] employed long short-term memory recurrent neural networks to forecast influenza trends from multiple data sources, including virologic surveillance, influenza geographic spread, Google trends, climate and air pollution; their results also exhibited high prediction accuracy.

Although it is known that many diseases are related to food contamination, studies on their correlations are relatively few, mainly because the number of food contaminations is much larger than the number of air pollutants, and thus classical regression methods and shallow ANNs become inefficient in handling complex correlations in such a high-dimensional feature space. Recently, deep neural networks (DNNs) are a powerful tool for modeling complex probabilistic distributions over a large number of influence factors by automatically discovering intermediate abstractions, layer by layer. Song et al. [21] developed a DNN based on a denoising autoencoder [22] to predict gastrointestinal-infection morbidity from food-contamination data in four counties in China during 2015–2016, and the results showed that the deep-learning model had significantly higher prediction accuracy than shallow ANNs. However, their work only concerned the morbidity of all acute gastrointestinal infections, i.e., it neither considered other gastrointestinal diseases such as chronic gastritis and gastrointestinal tumors, nor did it differentiate the morbidities of different gastrointestinal infections, such as acute gastritis and dysentery.

This study investigates the effects of food contamination on six main gastrointestinal diseases, acute gastroenteritis, chronic gastroenteritis, gastrointestinal ulcers, gastrointestinal tumors, food poisoning, and other acute gastrointestinal infections. We employed five methods, multiple linear regression (MLR), a three-layer feed-forward ANN, a deep belief network (DBN) [23], a deep autoencoder (DAE), and a deep denoising autoencoder (DDAE) [22], for correlation analysis and gastrointestinal-morbidity prediction. For each of the last three deep-learning methods, we respectively constructed two models, one using the basic gradient-based training algorithm and the other using an evolutionary training algorithm. Results showed that the deep-learning models achieved significantly higher accuracies than the MLR and shallow ANN models, and the DDAE with evolutionary training exhibited the highest prediction accuracy.

## 2. Materials and Methods

### 2.1. Materials

We collected data from ten cities/counties in central China, Yichun City (Yuanzhou Municipal District), Gao’an City, Wanzai County, Tonggu County, Pingxiang City (Anyuan Municipal District), Shangli County, Ji’an County, Xingan County, Liling City, and Chaling County, from May 2015 to September 2018 (178 weeks). These cities/counties have similar dietary habits and levels of health services. The dataset consists of two parts:

- Weekly food-contamination data from food-supervision departments. They include 119 types of food (given in Table 1) and 227 types of contaminants (given in Table 2). Therefore, the total number of contaminant indicators was at most 27,013. However, in practice, it is impossible to inspect so many contaminants, and thus the data tuples contain a large portion of missing values, and the average number of indicators per tuple is only approximately 4955.
- Weekly gastrointestinal-morbidity data from hospitals and healthcare-management departments. As aforementioned, these involve six general types of gastrointestinal diseases.

We constructed a data tuple per week for each city/county; the total number of tuples is 1780. If an indicator was measured more than once in a week, we took the mean value in the tuple.

### 2.2. Methods

We used eight machine-learning models for gastrointestinal-morbidity prediction based on food contamination. The aim of model training was to minimize the root mean squared error (RMSE) between the actual model outputs and the expected outputs over the training set:

(1) $$\min\;\mathrm{RMSE}=\sqrt{\frac{1}{N}\sum_{i=1}^{N}{\left|y_{i}-{\hat{y}}_{i}\right|}^{2}}$$

where *N* is the number of tuples in the training set, $y_{i}$ is the model actual output of the *i*-th tuple, and ${\hat{y}}_{i}$ is the expected (labeled) output of the *i*-th tuple. In this study, the output morbidity $y_{i}$ is calculated as the ratio of the incidences to the resident population in the investigated region (the floating population is not taken into account because of the difficulty of data collection).

A model is evaluated based on its prediction accuracy over the test set. We used fivefold cross-validation, i.e., we partitioned the dataset into five equal-size pieces, and ran the validation five times, each using four pieces as the training set and the remaining piece as the test set. Prediction accuracy was averaged over the five validations.

#### 2.2.1. Multiple Linear Regression (MLR)

The MLR method calculates an output *y* from an *n*-dimensional input x as:

(2) $$y=a_{0}+\sum_{i=1}^{n}a_{i}x_{i}$$

where $a_{i}$ are the regression coefficients (i=1,2,…,n). Here, *n* = 27,013; if a value $x_{i}$ is missing, it is filled by the mean value of those nonmissing $x_{i}$ of training tuples.

#### 2.2.2. Shallow Neural Network

We used a three-layer feed-forward ANN trained by the back-propagation algorithm. Each neuron in the input layer directly accepts an input component $x_{i}$, while each neuron *j* in the hidden layer calculates an inner output $z_{j}$ as:

(3) $$z_{j}=s\left(\sum_{i=1}^{n}w_{ij}x_{i}-\theta_{j}\right)$$

where $\theta_{j}$ is the threshold of the neuron, $w_{ij}$ is the connection weight between the *i*-th input neuron to the neuron *j*, and *s* is the sigmoid activation function:

(4) $$s\left(u\right)=\frac{1}{1+e^{-u}}$$

Similarly, the output neuron calculates the final output *y* as:

(5) $$y=s\left(\sum_{j=1}^{m}w_{j}z_{j}-\theta_{0}\right)$$

Empirically, we set number of neurons *m* in the hidden layer to $\sqrt{n}$.

#### 2.2.3. Deep Belief Network (DBN)

A DBN [23] consists of a stack of Restricted Boltzmann Machines (RBMs) [27]. An RBM, consisting of a visible input layer and a hidden layer, is an energy-based probabilistic model that defines a joint probability distribution over an input vector x and a hidden vector z as:

(6) $$P\left(x,z\right)=\frac{1}{\sum_{i=1}^{N}\exp\left(-E\left(x_{i},z_{i}\right)\right)}\exp\left(-E\left(x,z\right)\right)$$

where $E\left(x,z\right)=-x^{T}bx-z^{T}cz-x^{T}wz$, and b, c, and w are the parameter vectors representing visible-to-visible, hidden-to-hidden, and visible-to-hidden interaction weights, respectively. Note that a basic RBM learns distributions over binary vectors, but we can use Gaussian-Bernoulli energy function to transform a real vector into a binary one [28], and then use DBN to learn distributions over the transformed binary vector [29].

After fine-tuning the structural parameters of the DBN on the training sets, we set the number of hidden layers to four, and set the numbers of neurons in the hidden layers to 3860, 550, 80, and 12, respectively. A Gaussian mixture model was added to the topmost RBM of DBN to produce output morbidity *y* from topmost hidden vector z. DBN training consists of two stages. The first stage is pretraining, which tries to maximize the joint distribution of each RBM over the training set layer-by-layer:

(7) $$\underset{b,c,w}{\arg\;\max}J=\frac{1}{N}\sum_{i=1}^{N}\log P\left(x_{i},z_{i}\right)$$

The second stage is to minimize the RMSE of the whole DBN over the training set.

#### 2.2.4. Evolutionary Deep Belief Network (EvoDBN)

A classical DBN is trained by a gradient-based, layerwise training algorithm [30], which is easily trapped in local optima, especially when the dimension is high. This issue can be tackled by using evolutionary training algorithms, which evolve populations of solutions to simultaneously explore multiple regions in the solution space to increase the chances of jumping out of local optima [31]. Here, we employed a recent efficient evolutionary algorithm called water wave optimization (WWO) [32], which has exhibited competitive performance compared to many other popular evolutionary algorithms in neural-network training [33].

To solve an optimization problem, WWO evolves a population of candidate solutions by mimicking wave propagation and breaking in shallow water. In WWO, each solution *X* is analogous to a wave. The higher the energy (fitness) f(X), the smaller the wavelength $\lambda_{X}$, and thus the smaller the range that the wave propagates. $\lambda_{X}$ is initially set to 0.5, and then updated at each generation as:

(8) $$\lambda_{X}=\lambda_{X}\cdot \alpha^{-\left(f\left(X\right)-f_{\min}+\epsilon \right)/\left(f_{\max}-f_{\min}+\epsilon \right)}$$

where $f_{\max}$ and $f_{\min}$ are the maximum and minimum fitness among the population, respectively, α is the wavelength-reduction coefficient suggested set to 1.0026, and ϵ is a very small number to avoid division by zero. At each generation, *X* is propagated by adding an offset proportional to $\lambda_{X}$ to each dimension $X_{i}$ as follows:

(9) $$X_{i}^{\prime }=X_{i}+\lambda_{X}\cdot \mathrm{rand}\left(-1,1\right)\cdot L_{i}$$

where $L_{i}$ is the length of the *i*-th dimension of the solution space. Whenever a propagation produces a new best solution $X^{*}$, it is broken into several solitary waves, each of which moves a small distance from $X^{*}$ in a random dimension *i*:

(10) $$X_{i}^{\prime }=X_{i}^{*}+N\left(0,1\right)\cdot \beta L_{i}$$

where β is the breaking coefficient, and N denotes a normal distribution. The best solitary wave, if better than $X^{*}$, replaces $X^{*}$ in the population.

The EvoDBN uses the same architecture as DBN, and also employs a Gaussian mixture model to produce output morbidity. When training EvoDBN, WWO is first applied to optimize the {b,c,w} parameters of each RBM layer by layer, where f(X) corresponds to the objective function in Equation (7). After pretraining, WWO is applied to optimize the parameters of the DBN as a whole, where f(X) is inversely proportional to RMSE.

#### 2.2.5. Deep Autoencoder (DAE)

An autoencoder also consist of a visible input layer (called an encoder) and a hidden layer (called a decoder). It first transforms (encodes) an input vector x to a hidden representation z through affine mapping

(11) z=s(wx+b)

and then maps (decodes) z back to a reconstructed vector $x^{\prime }$ in the input space:

(12) $$x^{\prime }=s\left(w^{\prime }z+b^{\prime }\right)$$

The aim of autoencoder training is to minimize the average reconstruction error over the training set:

(13) $$\underset{w,b,w^{\prime },b^{\prime }}{\arg\;\min}L=\frac{1}{N}\sum_{i=1}^{N}{∥x,x^{\prime }∥}^{2}$$

A DAE [23] consists of a stack of autoencoders. Its training consists of two stages. The first stage is to train each autoencoder layer by layer, and the second stage is to train the whole DAE to minimize the RMSE over the training set.

For the morbidity-prediction problem, we used a DAE with four hidden layers, and tuned the numbers of neurons in the hidden layers to 4500, 640, 80, and 12, respectively. It also employed a Gaussian mixture model to produce output morbidity.

#### 2.2.6. Evolutionary DAE (EvoDAE)

Similarly, we implemented a DAE trained by the WWO evolutionary algorithm, which is first applied to minimize the reconstruction error in Equation (13) of each autoencoder layer by layer, and then applied to minimize the RMSE of the whole DAE. The EvoDAE uses the same structure (including the top-level Gaussian mixture model) as DAE.

#### 2.2.7. Deep Denoising Autoencoder (DDAE)

A denoising autoencoder is a variant of the basic autoencoder. It first randomly adds some noise to an initial input vector x to form a corrupted $\tilde{x}$, and then encodes $\tilde{x}$ to a hidden representation z, which is then decoded to a reconstructed $x^{\prime }$. The aim of denoising-autoencoder training is to reconstruct a clean “repaired” $x^{\prime }$ from a corrupted $\tilde{x}$, which can still be represented by Equation (13). The key difference is that z is deterministic mapping of $\tilde{x}$ and thus the result of a stochastic mapping of x.

Similarly, a DDAE [22] consists of a stack of denoising autoencoders. Its training consists of two stages. The first stage is to train each denoising autoencoder layer by layer, and the second stage is to train the whole DDAE to minimize the RMSE over the training set. For our prediction problem, the DDAE model uses the same structure (including the top-level Gaussian mixture model) as DAE.

#### 2.2.8. Evolutionary DDAE (EvoDDAE)

Similarly, we implemented a DDAE trained by the WWO evolutionary algorithm, which is first applied to minimize the reconstruction error of each denoising autoencoder layer by layer, and then applied to minimize the RMSE of the whole DDAE. The EvoDDAE model uses the same structure as DDAE.

## 3. Results

**Figure 1:**
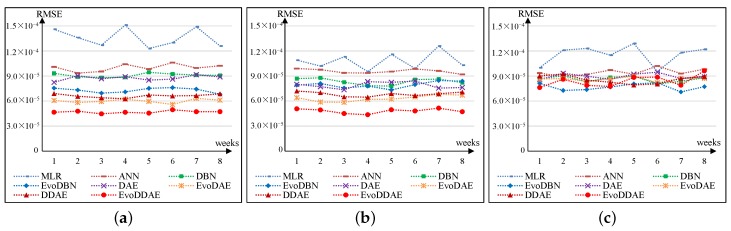
Prediction root mean squared error (RMSE) of the eight models using different time lags. *x*-axis denotes the time lag in weeks, and *y*-axis denotes the RMSE. (**a**) Chronic gastroenteritis; (**b**) gastrointestinal ulcers; (**c**) gastrointestinal tumors. MLR: multiple linear regression; ANN: artificial neural network; DBN: deep belief network; EvoDBN: evolutionary DBN; DAE: deep autoencoder; EvoDAE: evolutionary DAE; DDAE: deep denoising autoencoder; EvoDDAE: evolutionary DDAE.

According to historical experience, the weekly morbidities of acute gastroenteritis, food poisoning, and other acute gastrointestinal infections are predicted based on food-contamination data one week before. However, the time-lag effects of food contamination on chronic gastroenteritis, gastrointestinal ulcers, and gastrointestinal tumors are unknown. Therefore, we first tested the RMSE of the models for predicting the morbidities of the three types of diseases with a time lag of 1–8 weeks, respectively. Results are given in Figure 1, from which we can observe that:

- For chronic gastroenteritis, ANN and EvoDAE achieved the best RMSE when the lag was 2–3 weeks; DBN, EvoDBN, and DDAE achieved the best RMSE when lag was 3–4 weeks; DAE achieved the best RMSE when lag was 5–6 weeks; EvoDDAE achieved the best RMSE when lag was 3–5 weeks; and MLR showed good performance when lag was 3, 5, or 8 weeks (more irregular than other models).
- For gastrointestinal ulcers, ANN, DDAE, and EvoDDAE achieved the best RMSE when lag was 3–4 weeks; DAE and EvoDAE achieved the best RMSE when lag was 2–3 weeks; DBN achieved the best RMSE when lag was 4–5 weeks; EvoDBN achieved the best RMSE when lag was 3–5 weeks; and MLR showed good performance when lag was 4 or 6 weeks.
- For gastrointestinal tumors, the time-lag effect greatly varied among the models.

Consequently, we chose a time lag of three weeks for predicting the morbidities of both chronic gastroenteritis and gastrointestinal ulcers. For gastrointestinal tumors, because we could not determine an appropriate time lag for most models, we determined a different time lag for each model that resulted in the best RMSE for the model (6, 2, 6, 2, 1, 6, 5, and 1 week(s) for MLR, ANN, DBN, EvoDBN, DAE, EvoDAE, DDAE, and EvoDDAE, respectively).

**Figure 2:**
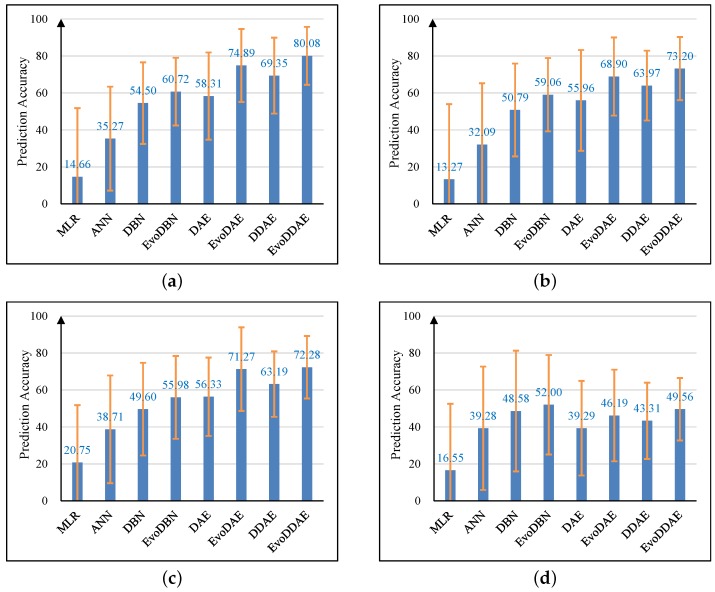

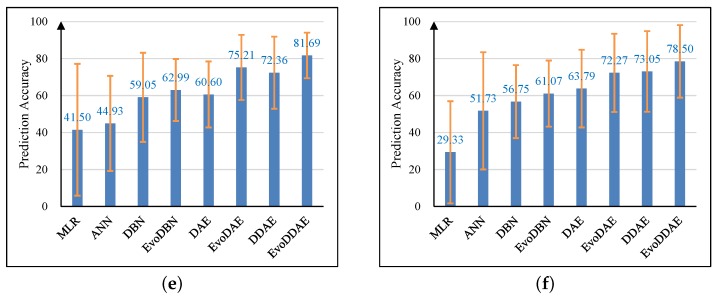
Accuracies of the models for gastrointestinal morbidity prediction. (**a**) Acute gastroenteritis; (**b**) chronic gastroenteritis; (**c**) gastrointestinal ulcers; (**d**) gastrointestinal tumors; (**e**) food poisoning; (**f**) other gastrointestinal infections.

Figure 2a–f presents the prediction accuracies of the models for the six gastrointestinal diseases, respectively. Results show that the traditional MLR exhibits the worst prediction performance on all diseases, the shallow ANN exhibits significantly better performance than MLR, and all deep-learning models exhibited much better performance than the MLR and shallow ANN. Among the six deep models, EvoDDAE exhibited the best performance on five diseases except gastrointestinal tumors. The average prediction accuracy of EvoDDAE was over 80% on acute gastroenteritis and food poisoning, close to 80% on other gastrointestinal infections, and approximately 72%–73% on chronic gastroenteritis and gastrointestinal ulcers. For gastrointestinal tumors, except that EvoDBN obtained an average perdition accuracy of approximately 52%, the accuracies of all other models were less than 50%, which indicates that the gastrointestinal-tumor morbidity is difficult to predict using these models. We also observed that, in most cases, the performance of a deep model could be significantly improved by using evolutionary training to replace traditional gradient-based training.

## 4. Discussion

This study constructed and compared eight models for predicting the morbidities of six main gastrointestinal diseases from food contamination. Results demonstrate that some deep-learning models can achieve relatively high prediction accuracy. However, this does not mean that gastrointestinal diseases are mainly caused by food contamination, or that gastrointestinal morbidities in a region are mainly determined by the levels of food contamination. In fact, the relationships between food contamination and gastrointestinal morbidities can be highly complex and probabilistic, and morbidities are also affected by many other factors, such as the dietary habits and working pressures of inhabitants, and the levels of health services of that society. Our study reveals that, given a large number of historical data of food contamination and gastrointestinal morbidities in a region, we could use deep neural networks to learn such highly complex and probabilistic relationships. After sufficient training, we could obtain models that embed other influencing factors into model parameters, and thus output relatively accurate morbidities from food-contamination inputs. Consequently, the prediction results would be very useful to improve healthcare services.

In general, the traditional MLR model is incapable of learning complex relationships for morbidity prediction. According to our results, its average prediction accuracy is below 20% on most diseases. For food poisoning, MLR achieves the highest prediction accuracy of 41.5%, which is also significantly less than the seven other models. The low performance of MLR indicates that relationships between food contamination and gastrointestinal morbidities are highly nonlinear and probabilistic, which is beyond the capability of the linear model.

The shallow ANN model performs much better in approximating nonlinear relationships. However, its average prediction accuracy is only between 30% and 40% in most cases, which is still too low for medical management. This is mainly because the number of food-contamination indicators is large, and the generalization ability of the classical three-layer structure of ANN decreases dramatically with increasing dimension.

DNN models can effectively overcome the limitations of the MLR and shallow ANN models, as they can learn complex probabilistic distributions over a large number of influence factors by automatically discovering intermediate abstractions layer by layer. Comparing DBN and DAE, two of the most widely used DNNs, DAE achieved higher accuracies than DBN on five gastrointestinal diseases, while DBN only achieved higher accuracy on gastrointestinal tumors. This indicates that the energy-based probabilistic model of DBN is less effective than the reconstruction-error minimization model of DAE in morbidity prediction. By introducing the denoising learning mechanism into DAE, DDAE achieved significantly higher accuracies than DBN and DAE on all gastrointestinal diseases. This is because the food-contamination data inevitably contain much noise, which can often mislead the learning process of DAE, while DDAE is much more robust in handling noisy inputs.

It was also observed that the prediction performance of all three DDNs could be significantly improved by equipping them with evolutionary training algorithms, because gradient-based training algorithms are easily trapped in local optima. An evolutionary algorithm uses a population of candidate solutions to simultaneously explore the search space; if some solutions are trapped in local optima, others can still explore other regions and help the trapped solutions jump out of local optima. Consequently, evolutionary DNNs can effectively suppress premature convergence and exhibit high learning abilities. Among the eight models, EvoDDAE that combines DDAE with evolutionary learning exhibited the best performance for morbidity prediction.

Among the six main types of gastrointestinal diseases, the prediction accuracies on three types of acute diseases are generally higher than other diseases, because the pathogenic mechanisms of acute diseases are relatively simpler, and their time-lag effects are easier to determine. That is why all models achieved the highest prediction accuracies on food poisoning, which is considered as “the most acute” disease. Among the diseases, each DNN model achieved the lowest prediction accuracy on gastrointestinal tumors, mainly because the pathogenic mechanisms of tumors are more complex than other diseases, and thus their correlation with food contamination is much weaker or is much difficult to learn.

## 5. Conclusions

This study compared eight machine-learning models for predicting the morbidities of six main gastrointestinal diseases from food-contamination data. Experiments on the datasets from ten cities/counties in central China demonstrate that the DNN models achieved significantly higher accuracies than the classical MLR and shallow ANN models, and the DDAE model with evolutionary learning exhibited the best prediction performance. Results also indicate that model accuracies are generally higher on acute gastrointestinal diseases than on other diseases, but it is difficult to predict the morbidities of gastrointestinal tumors. Moreover, a drawback of DNN models is that it takes significant effort to tune the structural parameters of the networks.

The studied deep-learning models could be utilized for the morbidity prediction of many other diseases whose influencing factors are large and complex. However, DNNs typically need to be trained on a large amount of labeled data, but disease- and health-related data are often very limited. Thus, we are currently studying unsupervised and transfer-learning technologies [34] for adapting the models from some well-known diseases to other diseases with insufficient data. Our future work also includes integrating the deep-learning models with fuzzy systems to handle uncertain information in the data [35,36], and utilizing the morbidity-prediction results for improving medical services, such as for medical-resource preparation and drug-procurement planning [37]. We believe that the combination of emerging deep-learning and intelligent decision-making technologies can significantly improve our society’s healthcare services.

## Figures and Tables

**Table 1:** Types of food for morbidity prediction [21].

Class	Food
Cereals	Rice, wheat, barley, corn, millet, black rice, sticky rice
Beans	Soybean, mung soybean, red bean, black bean, broad bean, pea, cow pea, hyacinth bean, kidney bean, sword bead
Vegetables	Cabbage, pak choi cabbage, baby cabbage, celery cabbage, celery, lettuce, broccoli, Chinese broccoli, mustard leaf, leaf lettuce, okra, rape, spinach, water spinach, potherb mustard, amaranth, cauliflower, purslane, yam, carrot, celtuce, summer radish, loofah, tomato, cucumbers, lappa, radish, potato, sweet potato, pumpkin, bitter gourd, white gourd, chilli pepper, bell pepper, green pepper, sweet pepper, pod pepper, pea sprout, soybean sprout, mung bean sprout, Chinese toon sprout, shiitake, button mushroom, oyster mushroom, needle mushroom, agaric, day lily, tremella, spring onion, Chinese onion, ginger, caraway, garlic, fragrant-flowered garlic, garlic sprouts
Fruits	Apple, gala apple, bergamot pear, snow pear, mili pear, pineapple, orange, navel orange, vibrio mimicus, pomelo, peach, nectarine, melon, watermelon, Hami melon, apricot, plum, cherry, bayberry, grape, longan, lychee, winter jujube, red jujube, sugarcane, pitaya
Meals and eggs	Pork, beef, mutton, chicken, duck, egg, duck egg, quail egg
Aquatic	Kelp, laver, carp, grass carp, yellow croaker, perch, crucian, prawn, river prawn, crab, river crab, river snail

**Table 2:** All 227 contaminants used for morbidity prediction [21].

Class	Subclass	Contaminants
Inorganic contaminants	Heavy metals	Pb, Cd, Hg, Cu, Ni, As, Be, Bi, Sb, Tl, Cr, Mo, Ni, Zn, F, V
	Others	cyanide, nitrate, nitrite, sulfate, carbonate
Organic contaminants	Hydrocarbons	benzene series, polycyclic aromatic hydrocarbons, total petroleum hydrocarbon
	Halogenated	hydrochlorofluorocarbons, chlorinated solvents, polychlorinated biphenyls, dioxin
	Oxygenated	alcohols, phenols, ethers, esters, phthalate
	Dyes	Azo, quaternary ammoniun compounds, benzidine, naphthylamine
	Plastics	polypropylene, polyphenyl ether, polystyrene, phthalic acid esters
	Pesticides	66 commonly used pesticides [24]
	Herbicides	18 commonly used herbicides [25]
	Endocrine disruptors	68 chemicals [26]
	Others	trichloroethylene, organochlorine pesticide
Pathogenic organisms	Bacteria	salmonella, shigella, dysentery bacillus, plague bacillus, tubercle bacillus, typhoid bacillus, diphtheria bacillus, Francisella tularensis, Brucella, vibrio parahaemolyticus, vibrio cholerae, vibrio mimicus, vibrio fluvialis, clostridium tetani, clostridium botulinum, clostridium perfringens, staphylococcus aureus, Bacillus anthraci, Escherichia coli, Yersinia, helicobcter pylori, campylobacter jejuni, aeromonas hydrophila, roundworm eggs, hookworm eggs
	Fungi	candida albicans, aspergillus fumigatus, mucor racemosus
	Virus	rotavirusm, norovirus, sapovirus, astrovirus

## Abbreviations

The following abbreviations are used in this manuscript:

ANN	Artificial neural network
DNN	Deep neural network
MLR	Multiple linear regression
RMSE	Root mean squared error
DBN	Deep belief network
RBM	Restricted Boltzmann machine
DAE	Deep autoencoder
DDAE	Deep denoising autoencoder
WWO	Water wave optimization
